# Starting points for the development of new targeted therapies for glioblastoma multiforme

**DOI:** 10.1016/j.tranon.2024.102187

**Published:** 2024-11-11

**Authors:** Agnieszka Rusak, Benita Wiatrak, Klaudia Krawczyńska, Tomasz Górnicki, Karol Zagórski, Łukasz Zadka, Wojciech Fortuna

**Affiliations:** a Division of Histology and Embryology, Department of Human Morphology and Embryology, Faculty of Medicine, Wroclaw Medical University, T. Chalubinskiego 6a St., Wroclaw 50-368, Poland; b Department of Pharmacology, Faculty of Medicine, J. Mikulicza-Radeckiego 2 Street, Wroclaw 50-345, Poland; c Division of Ultrastructural Research, Wroclaw Medical University, T. Chalubinskiego 6a St., Wroclaw 50-368, Poland; d Department of Clinical Pharmacology, Wroclaw Medical University, Borowska 211a, Wroclaw 50-556, Poland; e Department of Neurosurgery, Wroclaw Medical University, Borowska 213St, Wroclaw 50-556, Poland

**Keywords:** Glioblastoma, Targeted therapy, Chemoresistance phenotype, Anticancer treatment

## Abstract

Glioblastoma multiforme (GBM) is one of the most aggressive and lethal brain tumors, characterized by rapid growth, invasiveness, and resistance to standard therapies, including surgery, chemotherapy, and radiotherapy. Despite advances in treatment, GBM remains highly resistant due to its complex molecular mechanisms, including angiogenesis, invasion, immune modulation, and lipid metabolism dysregulation. This review explores recent breakthroughs in targeted therapies, focusing on innovative drug carriers such as nanoparticles and liposomes, and their potential to overcome GBM's chemo- and radioresistant phenotypes. We also discuss the molecular pathways involved in GBM progression and the latest therapeutic strategies, including immunotherapy and precision medicine approaches, which hold promise for improving clinical outcomes. The review highlights the importance of understanding GBM's genetic and molecular heterogeneity to develop more effective, personalized treatment protocols aimed at increasing survival rates and enhancing the quality of life for GBM patients.

## Highlights

• Glioblastoma multiforme is one of the most aggresive tumours with poor prognosis for patients.
• Standard therapy has still based on a combination therapy of surgical resection, temozolomide and radiotherapy but clinical lack of response is prelevant.
• New formulations of drug carriers such as liposomes, nanoparticles and polymers have been intensively explored to improve clinical outcomes in glioblastoma.
• Targeted therapy based on new drug formulations or advanced carrier drugs based on a thorough understanding of glioblastoma pathology could bring the expected breakthrough in clinical therapy.

## Graphical abstract

**Figure:**
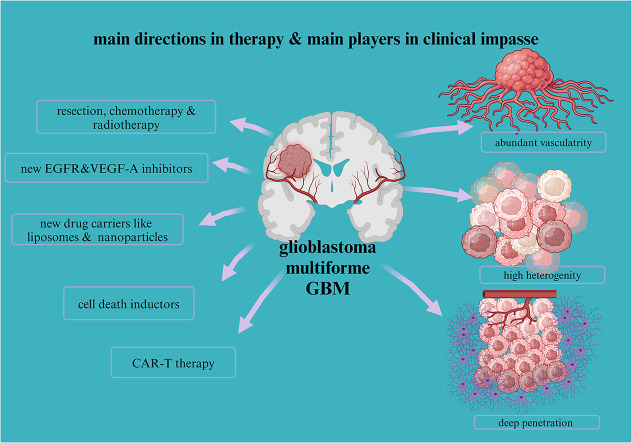

## Introduction

Glioblastoma multiforme (GBM), a grade IV tumor according to the WHO classification, is the most aggressive subtype, known for its rapid proliferation, invasiveness, and poor prognosis despite available treatment strategies [1]. The annual incidence of GBM is about 2–3 new cases per 100,000 people, representing 15–20 % of all primary brain tumors, and primarily affects older adults, with a median age of 64 years at the time of diagnosis [2]. Despite multimodal treatment, including surgery, radiotherapy, and chemotherapy, the median survival time for patients is only 12 to 15 months [3].

The classification of GBM into molecular subtypes—classical, proneural, and mesenchymal—further complicates the disease's landscape [4]. For example, the classical subtype is associated with EGFR amplification, while proneural gliomas are usually linked to mutations in the *IDH1* and *PDGFRA* genes [5]. The mesenchymal subtype, characterized by *NF1* mutations and activation of the NF-κB pathway, has the worst prognosis [5]. Understanding these molecular differences is crucial for developing personalized therapies [6].

**Fig. 1:**
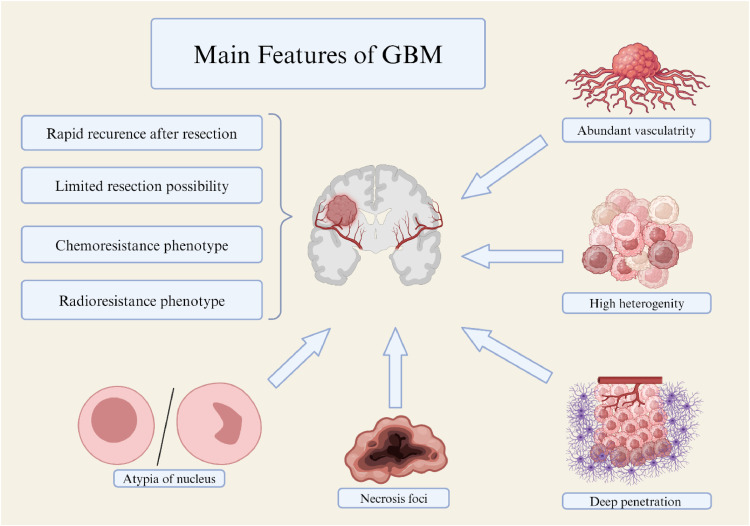
Characteristic features of glioblastoma multiforme (GBM) biology linked with treatment failure. Created with BioRender.com.

From a genetic perspective, key mutations in genes such as *PTEN, TP53*, and *EGFR* drive the progression of GBM [7]. Specific changes, such as *MGMT* promoter methylation, influence treatment response, particularly to alkylating agents like temozolomide (TMZ) [8]. Additionally, mutations in the *IDH1* gene, common in lower-grade gliomas but also found in GBM, are associated with better treatment outcomes [9]. Typical morphological features include an increasing vascular network resembling glomeruli, necrotic foci, nuclear atypia and pleomorphism, abnormal mitotic figures, variable GFAP expression, and the presence of giant cells characteristic of giant cell glioma (Fig. 1).

Current treatment options for GBM are limited and include surgical resection, radiotherapy, and chemotherapy, with TMZ as the standard chemotherapeutic agent [10]. However, GBM's well-documented resistance to therapy, along with its heterogeneity and invasive nature, leads to frequent relapses and poor long-term outcomes [11]. New therapies, such as targeted treatments and immunotherapies, are being intensely studied, offering hope for future breakthroughs [12].

Despite these advances, the complexity of GBM, its genetic diversity, and treatment resistance underscore the need for the development of novel therapeutic strategies [13]. Future research must focus on improving the understanding of the molecular mechanisms driving GBM progression and overcoming barriers such as drug resistance and the challenges of crossing the blood-brain barrier [14].

### Genetic features

Specific genetic variants frequently associated with GBM, such as activating mutations in the *EGFR* gene, its amplifications and point mutations (e.g. *EGFRvIII*), are observed in a significant number of patients [6,7,15]. In addition, mutations in tumor suppressor genes such as *PTEN* and *TP53* are widespread and affect 30–40 % of patients [16,17]. Mutations in *IDH1* (isocitrate dehydrogenase), which are more common in low-grade astrocytic gliomas may be also present in GBM, which influence patient prognosis and response to treatment, where mutation in IDH1 give more favorable overall survival prognosis for patient compare to *IDH* wild type [6,9,15].

**Fig. 2:**
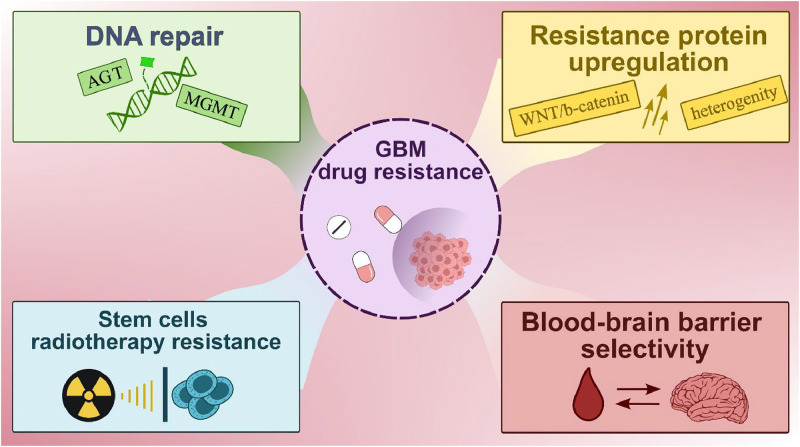
A key challenge in the treatment of GBM which may lead to clinical impairment in patients undergoing therapy. Created with BioRender.com.

Expression of *MGMT* (6 -methylguanine-DNA methyltransferase) gene, which is involved in DNA repair, affects treatment sensitivity, with methylation of its promoter region mostly increasing sensitivity to alkylating agents, such as TMZ [8,10]. Other gene mutations, including *PIK3CA, RB1, NF1* and *CDKN2A/B*, may also affect disease progression by activating signaling pathways such as PI3K/Akt/mTOR or disrupting cell cycle regulation [[18], [19], [20]]. Loss of function of tumor suppressor genes such as *RB1* and *NF1* can lead to uncontrolled cell division and faster disease progression [20,21]. Understanding the underlying genetic mutations and molecular pathways that drive tumor growth is critical to developing effective therapeutic strategies and improving patient outcomes. Recent literature which focuses on GBM therapy has emphasized the importance of personalized medicine approaches, targeted therapies, immunotherapies, and molecular profiling in tailoring treatment regimens for individual patients [12,22,23]. Advances in genome sequencing technology have enabled the identification of new therapeutic targets and the development of precision medicine approaches for GBM treatment, for example non-coding mutations with regulatory potential (like in the genes *DLX5, DLX6, FOXA1, ISL1)*, and also epigenetic changes like DNA methylation (in *SPINT2, GATA6, NEFM, CCNA1)* [15,24]. In addition, ongoing research is focused on overcoming challenges such as drug resistance, blood-brain barrier (BBB) penetration, and tumor heterogeneity to improve treatment efficacy and patient outcomes (Fig. 2) [14,25,26].

## Methods and concepts

This manuscript is a narrative review, aimed at summarizing current insights into the genetic landscape, treatment resistance, and potential therapeutic targets for glioblastoma multiforme (GBM). Our objective was to provide a comprehensive overview of the key pathways and molecular mechanisms involved in GBM, as well as to highlight new treatment avenues and challenges in overcoming resistance.

To select relevant literature for this review, we conducted a broad search across various databases, including PubMed, Scopus, and Web of Science, focusing on studies published between 2000 and 2023. Keywords used in our search strategy included "glioblastoma," "genetic mutations," "targeted therapy," "chemoresistance," and "treatment resistance, “temozolomide in glioblastoma", “radiotherapy in glioblastoma We also included recent studies discussing novel drug formulations, nanomedicine, and immunotherapies for GBM.

We applied inclusion criteria, which required studies to focus on genetic pathways, molecular targets, and new treatment strategies for glioblastoma. Exclusion criteria involved non-English studies and articles lacking relevance to GBM treatment.

## Current treatment for GBM

Radiotherapy is the standard adjuvant treatment after surgery or in cases where surgery is not possible. Its aim is to destroy remaining cancer cells and delay tumor growth [27,28]. Radiotherapy can be used as the primary form of therapy or in combination with chemotherapy. Chemotherapy plays a decisive role both in the initial treatment and in the treatment of recurrences [29,30]. The pharmacokinetics of chemotherapeutic agents current used in the treatment of GBM are described in Table 1.

**Table 1:** The pharmacokinetics of chemotherapeutic agents used in the treatment of GBM in terms of LADME (liberation, absorption, distribution, metabolism, excretion/elimination processes).

	Temozolomide (TMZ)	Lomustine (CCNU)	Procarbazine	Carmustine (BCNU)	Vincristine	References
**Administration**	oral (28-day cycles: 5 days drug and 23 day break)	oral administration (6-8 weeks cycles)	oral administration	intravenously	Intravenously	[[115], [116], [117], [118], [119]]
**Absorption and distribution**	■ prodrug, is quickly transformed into the active form (metabolite) in the body ■ this metabolite has the ability to cross the blood-brain barrier (BBB)	■ ability to penetrate the BBB ■ is well absorbed from the gastrointestinal tract	■ penetrates the BBB ■ is well absorbed from the gastrointestinal tract.	can cross the BBB, however its distribution in tumor tissue may vary and tumor concentrations may often be lower than in other tissues.	it is less effective at penetrating the BBB due to its chemical properties, which may limit its availability in tumor tissue in the brain; used in the treatment of GBM due to its antimitotic effect	[58,[120], [121], [122], [123], [124], [125], [126]]
**Half-life**	approximately 1–2 h	1–6 h (metabolites 16–48 h)	approximately 10 min	approximately 15–30 min	initial – 5 min, middle – 2.3 h, terminal – 85 h	[119,123, [127], [128], [129]]
**Metabolism**	metabolized by enzymes from the cytochrome P450 (CYP)	metabolized in the liver by microsomal enzymes	metabolized in the liver by oxidation into various active metabolites	metabolized in the liver by microsomal enzymes	metabolized in the liver by microsomal enzymes	[[130], [131], [132], [133], [134], [135], [136]]
**Elimination**	in the urine	mainly in the urine	in the urine	rapid elimination, primarily through excretion in the urine.	primarily through bile, with a minor proportion being excreted in urine	[119,123,127,128,133]

In recent years, immunotherapies have emerged as promising approaches that aim to harness the patient's immune system to combat neoplasm, although they are still in the clinical trial phase [31,32]. These treatment regimens may represent a new path for the treatment of GBM.

Supportive therapies, including symptomatic treatment, rehabilitation, palliative care and psychological support, are integral components of comprehensive care for neuro-oncological practice aimed at optimizing patient's quality of life [29,[33], [34], [35]]. Individualized treatment approaches are of paramount importance, taking into account factors such as the patient's age, general health, tumor location and stage and other relevant considerations [[36], [37], [38]].

### Alkylating agents

In the field of chemotherapy destined for GBM and other gliomas, several drugs play a crucial role in treatment regimens. TMZ, imidazotetrazole derivative of decarbazine, an orally administered DNA alkylating agent, is one of the most commonly used drugs. It disrupts the DNA double strands, inhibits their growth and triggers apoptosis. TMZ is often used as first-line therapy or in combination with radiotherapy and is a cornerstone in the treatment of newly diagnosed GBM. [[39], [40], [41], [42]]. TMZ is renowned for its enhanced RT, which is attributed to the rising incidence of DNA double-stranded breaking during the RT process. This synergy effect is particularly pronounced in tumours with MGMT-negative status, where the repair processes are diminished [43,44].

TMZ induces cell cycle arrest in the G2/M phase or apoptosis [40]. This compound can cross the blood-brain barrier and is spontaneously activated in acidic pH to monomethyltriazene 5-(3-methyltriazen-1-yl)-imidazole-4-carboxamide (MITC), which then undergoes a series of chemical transformations to form 5-aminoimidazole-4-carboxamide (AIC) and finally the methyl diazonium cation. This cation is involved in the methylation of DNA at the N7 and O6 positions of the guanines and also at the N3 regions of the adenines. This leads to cytotoxic effects due to the unrepaired O6 methylation of guanine (O6mG), which leads to breakage of the DNA double strands. For patients undergoing treatment, TMZ therapy is a standard regimen followed by radiotherapy, usually with a dose of 2 Gy per fraction administered on a patient-dependent schedule per day until the total dose reaches 60 Gy. With this standard regimen, clinical results can be achieved in 55 % of patients with glioblastoma (GBM). However, due to the DNA repair system methyl-guanine-methyl-transferase (MGMT), GBM can revert to a TMZ-resistant phenotype [10,40]. The second mechanism that counteracts the effect of TMZ in MMR (mismatch repair); both are described later in this article

Lomustine (CCNU) is another DNA-alkylating agent used in the treatment of various brain tumors, including GBM. By forming bonds with DNA through the transfer of alkyl groups, lomustine causes DNA damage and inhibits replication, ultimately leading to apoptosis of cancer cells [45,46].

Carmustine (BCNU), alkylating agents acts as an inhibitor of monoamine dehydrogenase, is also used to treat GBM. It inhibits DNA and protein synthesis and thus contributes to the inhibition of cancer cell growth. BCNU is administered by intravenous injection, either as monotherapy or in combination with other drugs [47,48]. BCNU is also available in the form of Gliadel wafers, implanted directly into the brain during surgical removal of the tumor [49,50]. Procarbazine, a monoamine oxidase inhibitor, is frequently used in complex chemotherapy protocols for GBM treatment, often in combination with drugs such as CCNU and vincristine. This combination therapy is particularly effective in the treatment of anaplastic gliomas [[51], [52], [53]].

### Mitosis inhibition

Vincristine presents a particular challenge due to its chemical properties, which may compromise its ability to effectively penetrate the BBB and distribute throughout brain tumor tissue. Due to its relatively high molecular weight and hydrophilic nature, vincristine has difficulty penetrating the BBB, which is primarily composed of lipid cell membranes [54,55]. Despite its hydrophilic nature, vincristine also exhibits some lipophilicity, which impairs its distribution. Excessive lipophilicity may lead to nonspecific distribution of the drug in tissues, which could compromise its efficacy against brain tumors [56,57]. Nevertheless, its antimitotic effect makes it suitable for inclusion in GBM therapeutic regimens in certain cases [58]. Vincristine, an agent that interferes with cancer cell division by inhibiting microtubules, is occasionally used in chemotherapy regimens for GBM. By destabilizing microtubules and disrupting mitotic function, vincristine triggers apoptosis of cancer cells [51,59]. Irinotecan, a topoisomerase I inhibitor, is sometimes used in the treatment of GBM, particularly in cases of relapse or treatment failure. By inhibiting topoisomerase I, irinotecan induces DNA damage and apoptosis of tumor cells [60,61]. Common combinations of chemotherapeutic agents used in the treatment of gliomas include TMZ with CCNU and vincristine, procarbazine with CCNU and vincristine, and TMZ with bevacizumab (BVC) [51,53,62,63].

### Antiangiogenic therapy

Bevacizumab (BVC) is a monoclonal antibody directed against vascular endothelial growth factor A (VEGF-A). Administration of BVC against VEGF-A has been shown to inhibit angiogenesis and consequently reduce vascular permeability [4]. However, the benefits of BVC administration in clinical tumor therapy vary. In GBM therapy, BVC is most commonly used as an additional treatment alongside standard chemotherapy with TMZ and radiotherapy [63,65,66].

In some GBM patients, treatment with BVC may lead to a temporary improvement in symptoms, but this effect is often short-lived as the disease subsequently relapses. While some clinical trials have shown benefits in symptom relief and disease stabilization, the overall survival of GBM patients has not been significantly prolonged by treatment with BVC [66,67]. Nevertheless, BVC continues to be used in selected GBM cases to improve patients' quality of life by reducing brain edema and alleviating neurological symptoms [64,66,68].

### Mutation status and clinical response to treatment

The choice of chemotherapy for glioblastoma multiforme (GBM) is increasingly influenced by molecular mutations, as new research on the molecular subtypes of GBM suggests that certain mutations may influence the efficacy of certain drugs [4,43,69,70]. For example, glioma patients with MGMT promoter methylation show increased sensitivity to TMZ compared to patients without MGMT methylation [[71], [72], [73], [74]].

The activity of MGMT has been shown to facilitate the repair of DNA lesions under certain physiological conditions. This is achieved by converting the DNA lesion O6-methylguanine to guanine and the methyl group to cysteine. This mechanism is present in the DNA damage caused by TMZ therapy, leading to a reduction in the efficacy of this treatment [75]. Consequently, the combination of MGMT inhibitors and TMZ has the potential to improve clinical outcomes. The effects of the novel MGMT inhibitor AA-CW236 have recently been investigated in a number of studies demonstrating its potential [75].

In addition, the level of MGMT expression may serve as a prognostic factor for the efficacy of TMZ treatment, with tumors with higher MGMT levels exhibiting lower sensitivity to alkylating agents [36]. In addition, the methylation status of the MGMT promoter, which influences MGMT activity, is a prognostic factor for a more effective response to TMZ.

Another mechanism that counteracts the activity of TMZ is MMR (mismatch repair). The MMR pathway is activated as a consequence of the formation of base pairs resulting from the methylation of guanine during TMZ action. In general, MMR fixes a second strand of DNA when one of the strands is methylated, acting through the proteins MSH2, MSH6, MLH1 and PMS2. These proteins bind to guanine residues that are in an unequal position [40]. In contrast to the methylated guanine (O6mG), the thymine is in the leading position, and the repetition of this mechanism is known asthe futile cycle of MMR [76].

This leads to the initiation of the DNA repair phase in the cell cycle. However, the differentiation between the parental and daughter DNA strands leads to futile repair, DNA damage and consequent elimination of the cell. If there is insufficient MMR activity, the mutations accumulate in the cells, which can increase the risk of cancer development [40].

To overcome the TMZ resistance generated by the MGMT mechanism, a dose-dense response (DDR) was performed in conjunction with TMZ treatment. However, this strategy did not improve patient outcomes [40]. During TMZ treatment, resistance and a side effect in the form of TMZ-induced mutation occurred regardless of the specific TMZ treatment regimen. Hypermutated glioblastoma tumors are associated with a poorer prognosis for patient survival. It is interesting to note that low-grade tumors are more frequently hypermutated than high-grade tumors [40].

The differences in MGMT and MMR play a role in the treatment of patients with TMZ. From a clinical perspective, glioblastoma may be TMZ-sensitive in the absence of MGMT expression, with MMR increasing the efficacy of eliminating cancer cells in a futile cycle. Conversely, TMZ-resistant GBM tumors express MGMT or have no MMR expression. This peculiarity of GBM biology can be exploited to increase the efficacy of TMZ through the use of various inhibitors [77].

Although MGMT is known to be the most important desentizing factor in GBM for TMZ-RT treatment, recent studies suggest that there are other options, such as retinoblastoma binding protein 4 (RBBP4), which may be involved in treatment resistance regardless of MGMT status [78].

Patients diagnosed with gliomas with mutations in the *IDH1* or *IDH2* gene generally have a more favorable prognosis and are more likely to be classified as lower grade glioma (e.g., grade II or III glioma). These patients may respond better to chemotherapy, including drugs used to treat low grade gliomas [[79], [80], [81], [82], [83]].

The enzymes of isocitrate dehydrogenase (IDH), especially IDH1 and IDH2, play a central role in metabolic processes, particularly in the Krebs cycle, where they enable the oxidative decarboxylation of isocitrate. They are also involved in glutamine metabolism, lipogenesis and redox regulation. *IDH* mutations have been observed in a number of cancers, including GBM, acute leukemia and chondrosarcoma. In GBM grade IV, the *IDH* mutation is observed in over 70 % of cases, while in the lowest grades it is present in over 80 % of cases.

The *IDH* mutation is associated with a more favorable prognosis for the patient compared to the *IDH* wild-type phenotype. It is also associated with the highest sensitivity to radiotherapy and chemotherapy [9,80].

The consequence of the mutation in the *IDH* structure is the replacement of arginine at position 132 (in *IDH1*) or 140 or 172 (in *IDH2*), which is crucial for isocitrate binding, with amino acids that have a lower affinity for isocitrate: histidine, lysine and cysteine. these amino acids lead to a reduction in the affinity of isocitrate, while the affinity for NADPH is increased. This is due to the biochemical inactivation of the enzyme. This leads to further changes in metabolic processes, such as the utilization of glucose and glutamine in the absence of the Krebs cycle. In gliomas, glutaminolysis plays a compensatory role in this context. The affinity of mutant *IDH* for NAPDH is associated with the accumulation of ROS and oxidative damage. In addition, the *IDH* mutation has been observed to hinder the DNA repair process, with the formation of d-2-HG (D-2-hydroxyglutarate) playing a role in the Krebs cycle. d-2-HG has been identified as an inhibitor of DNA repair enzymes, including ALKBH2/3 (alpha-ketoglutarate-dependent dioxygenase alkB homolog 2). It is noteworthy that *IDH*-mutated gliomas exhibit a distinct metabolic profile characterized by reduced glycolysis, which is a common metabolic pathway in tumors [9]. It is noteworthy that the *IDH* mutation may prove useful for the targeted therapy of GBM. The *IDH1* and *IDH2* mutation inhibitor vorasidenib has recently demonstrated its potential against GBM, as described by Mellinghoff [83].

EGFR amplification is characteristic of GBM, most commonly associated with the classical subtype, and is detected in almost 60 % of primary GBM patients and 8 % of patients with secondary GBM tumors [70]. EGFR is a member of the ERBB family, which consists of the receptors ERBB1-ERBB4 (also known as HER1-HER4). Mechanistically, phosphorylation of EGFR leads to dimerization and activation of the GRB2 (growth factor receptor bound protein 2), PI3k/Akt, c-Src/FAK, RAS/MAPK/ERK and JAK/STAT-3 signaling pathways, which play an important role in processes that promote tumor progression, such as invasion and metastasis, proliferation, apoptosis inhibition and glioma stem cells [70,84].

Mutations of *EGFR* lead to an activation of this receptor and thus to a constitutive activation of downstream signaling pathways in GBM cells. Some *EGFR* mutations are specific for different types of cancer and can serve as biomarkers. In glioblastoma, the most common mutation is the *EGFRvIII* mutation, which is described as a loss of amino acids 6–273 and association with a new glycine residue between 5 and 274 amino acids compared to wild-type *EGFR*. As a result, a tumor-specific and immunogenic epitope is created by this novel linkage to the extracellular domain of EGFR [85].

Other *EGRF* mutations are also observed in *EGFRvI* (N-terminal deletion), *EGFRvII* (deletion of exons 14–15), *EGFRvIII* (deletion of exons 2–7), *EGFRvIV* (deletion of exons 25–27), *EGFRvV* (deletion of exons 25–28) as well as *EGFRvII* and *EGFRvIII*. A typical mutation in non-small cell lung cancer, but not common in GBM, is L858R in exon 21 and an in-frame deletion in exon 19 [70]. In addition, point mutations in the *EGF* receptor occurred in 24 % of GBMs: R108 K, A289V/D/T, G598D.

The activation of EGFR III is typical for tumor cells and could therefore have potential for targeted therapies with EGRF inhibitors. Hovewer, its expression in GBM is heterogeneous, and furthermore, EGFRIII amplicons may not be available for EGFR inhibitors because EGFRIII expression in cells changes dynamically during and after treatment and inhibitors are only present in low levels in tumor tissue. Resistance to EGFR inhibitors therefore remains a challenge as there are relatively few successful clinical results [70,86,87]. Current clinical trials, including new EGFR inhibitors, are described below in this article. Of note,the selection of EGFR-amplified tumors is critical to verify EGFR or variant III (EGFRvIII) expression status for more precise therapy, and there is no consensus in this area [86]. Recently, studies by French et al. have shown that FISH together with RT-qPCR methods can be a useful tool to select patients with EGFR amplification [86].

Patients carrying these mutations may show an increased response to EGFR-targeted drugs such as tyrosine kinase inhibitors (e.g. gefitinib, erlotinib). However, the efficacy of these drugs may vary depending on the specific *EGFR* mutations and the presence of associated molecular aberrations [70,[88], [89], [90]].

Numerous other molecular mutations, such as in the *PTEN, TP53* and *PDGFRA* genes, may also be important in GBM treatment. Analysis of the molecular profile of the tumor can help in tailoring treatment and selecting an appropriate therapy, including chemotherapy [25,[91], [92], [93]].

**Fig. 3:**
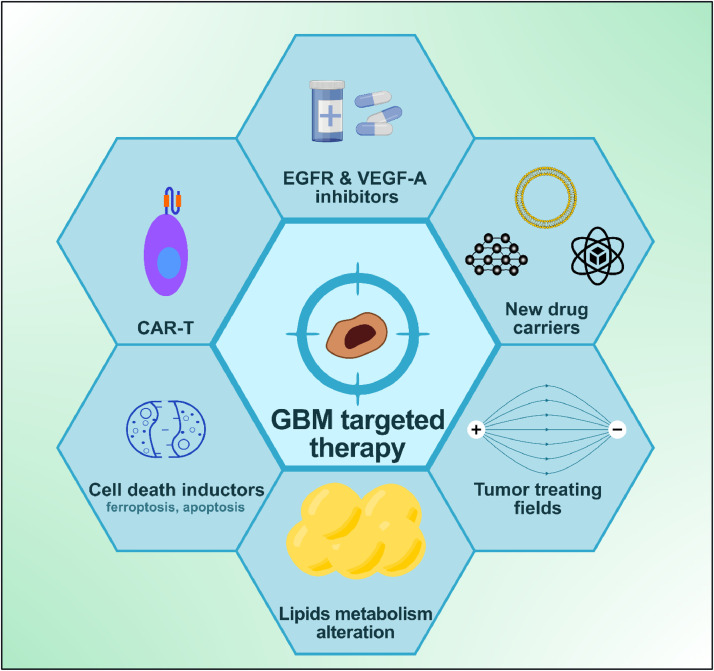
New avenues for targeted therapy design in GBM are currently under investigation and appear promising. Created with BioRender.com.

Despite progress, there are still gaps in the understanding of GBM, emphasizing the need for continued research and collaboration between stakeholders. Molecularly targeted therapies aim to target specific molecules or signaling pathways that are critical for cancer cell proliferation and survival [43,69,94]. Key points for targeted therapy was presented on Fig 3.

Examples include tyrosine kinase inhibitors such as EGFR or VEGF inhibitors, which may be indicated for certain glioma subtypes. Molecular profiling is crucial for tailoring therapeutic approaches for GBM [70,95].

The choice of therapy often depends on the molecular mutations of the tumor. Understanding the molecular makeup of the tumor helps predict the propensity for relapse and response to treatment [[96], [97], [98]]. Patients with *EGFR* mutations or *HER2* amplifications may benefit from targeted therapy with tyrosine kinase inhibitors such as erlotinib or lapatinib [70,[99], [100], [101]].

*MGMT* promoter methylation status determines sensitivity to alkylating agents [70,102,103].

*IDH1* mutations describe a different biology and prognosis and influence the choice of treatment [79,104]. TMZ-based chemotherapy may be more effective in patients with *IDH1* mutations [105]. Gene expression profiling facilitates the classification of molecular subtypes and aids in treatment prognosis and survival prediction [[106], [107], [108]]. Tumors with pathway mutations can be targeted with pathway inhibitors. Molecular techniques such as whole genome sequencing and gene expression profiling enable the identification of unique tumor characteristics and thus personalized therapy [97,109].

In clinical practice, molecular mutation analysis is increasingly being integrated into the management of GBM patients. The knowledge gained from these procedures allows physicians to better profiling neoplasms and tailor treatment to individual's needs [96,110]. However, ongoing research is focused on optimizing the use of this information to improve therapeutic outcomes in GBM patients [4,94,96]. Table 2 presents the current clinical trials in GBM, which also include the use of new inhibitors.

**Table 2:** Ongoing GBM clinical trials according to the clinicaltrials.gov database provided by the National Institutes of Health (NIH).

Treatment	Target	Status	Enrollment	Primary outcome measures	NCT number
Rindopepimut (CDX-110) with GM-CSF in combination with Bevacizumab	EGFRvIII	Completed, phase II	127	Progression-free survival (PFS), Objective Response Rate (ORR)	NCT01498328
Rindopepimut (CDX-110) with GM-CSF in combination with Temozolomide	EGFRvIII	Completed, phase III	745	Overall Survival (OS)	NCT01480479
SurVaxM Vaccine with Temozolomide	cells that express survivin	Active, not recruiting, phase II	66	PFS	NCT02455557
SurVaxM Vaccine with Temozolomide	cells that express survivin	Active, not recruiting, phase II	247	OS	NCT05163080
Anti-LAG-3 + Anti-PD-1	LAG-3, PD-1	Completed, phase I	63	Maximum tolerated dose (MTD)	NCT02658981
HER.CAR CMV-specific CTLs	HER2	Completed, phase I	16	Number of subjects with dose limiting toxicity after CTL infusion	NCT01109095
Epidermal growth factor receptor(EGFRv)III Chimeric antigen receptor (CAR) transduced PBL	EGFRvIII	Completed, phase I phase II	18	Number of treatment related adverse events, PFS	NCT01454596
IL13Ralpha2-CAR T cells	IL13Ralpha2	Active, not recruiting, phase I	82	Incidence of grade 3 toxicity, dose limiting toxicity (DLT)	NCT02208362
IL13Ralpha2-CAR T cells	IL13Ralpha2	Recruiting, phase I	30	Incidence of adverse events, OS	NCT04661384
B7-H3-targeting CAR-T cells	B7-H3 (CD276)	Recruiting, phase I	30	Incidence and severity of adverse events, OS	NCT05241392
B7-H3-targeting CAR-T cells	B7-H3 (CD276)	Recruiting, phase I phase II	40	Incidence and type of adverse events, MTD, PFS	NCT04077866
NK-92/5.28.*z* + Ezabenlimab	HER2	Active, not recruiting, phase I	42	Treatment-related adverse events, MTD, period of detectability of NK-92/5.28.z cells in blood and cerebrospinal fluid (CSF), cytokine profile in the blood and the CSF	NCT03383978
C225-ILs-dox	EGFR	Completed, phase I	9	Ratio of C225-ILs-dox concentration	NCT03603379
SGT-53	Delivery of the p53 cDNA to the tumor cells	Terminated, phase II	1	Tumor response	NCT02340156
Liposomal Curcumin (LC)	Rb, p53, MAPK, P13K/Akt, JAK/STAT, Shh, and NF-κB pathways	Recruiting, phase I phase II	30	The number of observed Dose Limiting Toxicity (DLTs)	NCT05768919
Verteporfin	mutant or amplified EGFR	Recruiting, phase I phase II	24	Incidence of adverse events (phase I), PFS, OS, response rate (RR) (phase II)	NCT04590664
NovoTTF-100A	antimitotic effect of tumor treating fields (TTF)	Completed, phase III	700	PFS	NCT00916409
ASC40 + Bevacizumab	fatty acid synthase (FASN)	Recruiting, phase III	180	PFS, OS	NCT05118776
ERAS-801	EGFR/ERBB1	Active, not recruiting, phase I	52	DLT, MTD, recommended dose (RD), adverse events	NCT05222802
Erlotinib	EGFR	Completed, phase I	22	Clinical Benefit Rate (either radiographic response or at least 6 months of progression-free survival)	NCT01257594
Epitinib Succinate (HMPL-813)	EGFR	Unknown, phase I	29	Objective Response Rate (ORR)	NCT03231501
Afatinib (BIBW 2992)	EGFR	Completed, phase I	36	DLT, MTD	NCT00977431
CART-EGFRvIII + Pembrolizumab	EGFRvIII	Completed, phase I	7	Number of subjects with treatment-related adverse events	NCT03726515
Peposertib	DNA-PK	Recruiting, phase I	29	MTD (stage I), ability of Peposertib (M3814) to cross the blood brain barrier (stage II)	NCT04555577
WP1066	STAT3 pathway	Recruiting, phase II	39	PFS, tumor microenvironment activation and cluster interactions	NCT05879250
BMS-986,205	IDO1	Active, not recruiting, phase I	18	Incidence of adverse events (AEs)	NCT04047706
Paxalisib (GDC-0084)	PI3K/mTOR	Completed, phase II	30	Dose limiting toxicities (DLTs)	NCT03522298
Selinexor	XPO1	Recruiting, phase I phase II	97	Recommended phase 2 dose (RP2D) (phase I), PFS	NCT05432804
Veliparib (ABT-888)	PARP-1, PARP-2	Active, not recruiting, phase II phase III	447	OS	NCT02152982
BGB-290	PARP-1, PARP-2	Recruiting, phase I	78	Proportion of participants with Dose Limiting Toxicities (DLTs)	NCT03749187
metformin (Glucophage)	Complex I in tumor mitochondria	Recruiting, phase II	640	PFS	NCT04945148

### Resistance to chemotherapy

The pharmacokinetics of chemotherapeutic agents used in GBM treatment are summarized in Table 1, where each drug is subject to LADME processes (Liberation, Absorption, Distribution, Metabolism, Excretion/elimination) that may vary depending on individual patient characteristics, disease state and drug dosage. Despite current therapeutic options, CNS tumors can develop resistance to the chemotherapeutic drugs commonly used in GBM treatment, such as TMZ, through various alterations in cell signaling pathways, including changes in DNA repair mechanisms [109,111,112]. Although these drugs can cross the BBB, their penetration may be limited, which may compromise treatment efficacy [25]. In addition, rapid metabolism and elimination from the body may reduce their therapeutic effect [113]. Given the great heterogeneity of GBM and its diverse cellular composition, the response to chemotherapeutic agents may vary from tumor region to tumor region, with some areas showing increased resistance [25,43,114].

## Therapy for GBM in clinical trials

The search for new treatment options for malignant brain tumors is necessary due to the low efficacy of the currently used methods, which include surgical resection, radiotherapy and chemotherapy. An important trend in the development of new GBM therapies is the use of immunotherapy, which recently appears to be the most promising therapeutic strategy in the oncology. Ongoing clinical trials on GBM with a focus on different targets are listed in Table 2.

To illustrate, CAR-T therapy has been approved for the treatment of patients with lymphoma and leukaemia (193). LAG-3 represents a promising target in melanoma and colorectal cancer (190). Monoclonal antibodies directed against PD-1 (pembolizumab, pembrolizumab, nivolumab) have demonstrated efficacy in the treatment of non-small cell lung carcinoma (NSCLC), small cell lung carcinoma (SCLC), oesophageal cancer and cervical cancer. The targeted PD-L1 (atezolizumab) demonstrated efficacy in TNBC (triple negative breast cancer) [137,138]. The therapy, which is targeted anti-PD-1/PD-L1 antibodies, has been approved as an immunotherapy for metastatic non-small cell lung cancer (NSCLC) patients [138].

The advantage of this form of therapy is that it targets exclusively neoplasm without damaging normal cells. It is currently being used in clinical trials focusing on anti-cancer vaccines, antibodies that block immune system checkpoints, T cells with chimeric antigen receptors (CAR-T) and natural killer (NK) cells.

### Cancer vaccines

Peptide vaccines against cancer acting by presenting tumor-related antigens for antigen-presenting cells, such as dendritic cells, to trigger an immune response against tumor. Recognition of the antigens as foreign by cytotoxic T lymphocytes leads to activation of these cells, resulting in elimination of the cancer cells. Amplification of the *EGFR* gene is frequently observed in solid tumors, including GBM. In tumors with *EGFR* gene amplification, a rearrangement often occurs, leading to the most common variant of the mutated extracellular domain, *EGFRvIII*. This variant lacks exons 2–7 and has a glycine residue at the junction of exons 1 and 8 [85]. This variant is not found in normal brain tissue, which is crucial for the development of a targeted therapy for this type of glioma. Currently, tumor-associated antigens or antigen-based vaccines are not used in the standard treatment of GBM. However, clinical trials are being conducted to investigate their safety and efficacy in the treatment of GBM.

The rindopepimut vaccine, also known as CDX-110, targets the surface antigen EGFRvIII. The ReACT phase II clinical trial investigated the effect of this vaccine in combination with the monoclonal antibody BVC (NCT01498328), which is currently used to treat GBM. This study analyzed a group of 73 patients (36 received rindopepimut, 37 were in the control group) and found that 80 % of patients treated with rindopepimut achieved high anti-EGFRvIII titers. These titers were associated with longer overall survival (OS) [139]. The efficacy of rindopepimut therapy was further validated in the phase III clinical trial ACT IV (NCT01480479). However, an interim analysis showed no significant clinical benefit of the vaccine, which led to the study being discontinued. The discrepancy between the results of ReACT and ACT IV may be due to the loss of EGFRvIII expression in recurrent tumors, which occurs in approximately 60 % of patients. This is a significant issue that limits the efficacy of immunotherapy in the treatment of GBM [140].

A phase II clinical trial was conducted with the vaccine SurVaxM, which consists of synthetic peptide antigens targeting the protein survivin expressed in GBM (NCT02455557). The translation results are 1652 out of 5000. Survivin, an apoptosis inhibitor, is overexpressed in cancer cells compared to normal cells and is involved in numerous signaling pathways related to tumor progression. These properties make survivin an attractive target for cancer therapies [141].The aim of the study was to investigate the safety, immunological effects and survival of patients with newly diagnosed GBM who received SurVaxM together with adjuvant TMZ after surgery and chemoradiotherapy. The study group comprised 64 patients, including 38 men and 26 women aged 20–82 years. Patients were administered four doses of SurVaxM subcutaneously, at a dose of 500 μg once every 2 weeks. The results of the study showed that the vaccine was safe and well tolerated by the patients. It also had a positive effect on progression-free survival (PFS) and OS by boosting the immune system's response. Six months after diagnosis, 60 % of all study participants were progression-free. The study found that the median progression-free survival (PFS) was 11.4 months and the median overall survival (OS) was 25.9 months after the first dose of SurVaxM [142]. A Phase II clinical trial (NCT05163080) is currently underway to determine whether the addition of SurVaxM to standard TMZ chemotherapy is more effective than treatment with TMZ alone in patients with newly diagnosed glioma.

### Antibodies that block checkpoints of the immune system

Antibodies that inhibit the checkpoints of the immune system constitute a growing trend in the development of cancer therapies. These antibodies are designed to prevent the formation of an immunosuppressive environment around the tumor and thus increase the effectiveness of the immune system in anticancer response. T lymphocytes have co-stimulatory molecules on their surface that have both activating and suppressive effects. These molecules act as checkpoints for the immune system. Ligands for these receptors are located on the surface of cancer cells. This allows the cancer cells to block the cytotoxic effects of T lymphocytes, which in turn allows the tumor to evade immune surveillance [143]. Inhibition of T lymphocyte activity is primarily achieved through activation of lymphocyte gene 3 (LAG-3), programmed death receptor 1 (PD-1) and cytotoxic T lymphocyte antigen 4 (CTLA-4) [144]. Antibody-mediated blockade of these receptors should restore function and increase proliferation of cytotoxic T lymphocytes, thereby enhancing the immune response in immunosuppressed diseases. Therefore, cancer immunotherapy that blocks immune checkpoints appears to be a promising direction for the development of cancer therapies. Current clinical trials are focused on determining the appropriate doses and administration regimens to maximize the efficacy of the therapy in treating cancer. The development of therapies based on antibodies that block checkpoints involves the combination with other therapeutics and the use of antibodies that block multiple checkpoints of the immune system.

A preclinical study in mice showed that blocking LAG-3 alone or in combination with an anti-PD-1 antibody led to a regression of gliomas [145]. The study also showed that LAG-3 is expressed in human gliomas, which served as the basis for subsequent clinical trials. A Phase I study was conducted in a group of patients with recurrent glioblastoma to evaluate the safety and effective dose of the anti-LAG-3 monoclonal antibody alone and in combination with the anti-PD1 antibody (nivolumab) (NCT02658981). Three doses of anti-LAG-3 were tested in the study: 80 mg, 160 mg and 800 mg. Anti-PD-1 was administered at a dose of 240 mg in combination with anti-LAG-3 at doses of 80 mg and 160 mg. It was found that the highest safe dose for anti-LAG-3 alone is 800 mg, and in combination with anti-PD-1, the doses are 160 mg for anti-LAG-3 and 240 mg for anti-PD-1 [146].

### CAR-T

Another promising approach in GBM therapy is adoptive cell therapy (ACT), a form of immunotherapy in which immune cells are in use. In ACT, cells are taken from the patient (autologous therapy) or a healthy donor (allogeneic therapy), cultivated ex vivo and then re-administered to the patient. It can be seen as a strategy for improving the immune response to various types of cancer. This therapy includes, among others: CAR-T involves genetically modifying T lymphocytes isolated from the patient's blood and attaching a chimeric antigen receptor (CAR) to their surface. These modified T lymphocytes are then administered to the patient, where the receptors on their surface bind to antigens on the surface of the cancer cells, leading to their elimination. This therapy specifically targets cancer cells and does not harm normal cells. CAR-T therapy is approved for the treatment of lymphoma and leukemia, where it has shown the greatest efficacy [147]. Although research suggests that it could also be used for solid tumors such as glioblastoma, no CAR-T-based therapy has yet been approved for this purpose. The development of this type of therapy is challenging due to the limited number of surface antigens that can be targeted by CAR-T and the high heterogeneity and antigenic variability of the tumor.

In the case of GBM, there is often overexpression of human epidermal growth factor receptor 2 (HER2), epidermal growth factor receptor variant III (EGFRvIII) and interleukin-13 receptor alpha 2 (IL13Rα2) [148]. Targeting these receptors has shown promising preclinical results, which led to further evaluation in clinical trials. In a phase I clinical trial (NCT01109095), the administration of autologous HER2 CAR-T cells to patients was found to have no adverse effects. Eight patients showed clinical benefit with a median overall survival of 24.5 months [149]. The administration of CAR-T cells targeting EGFRvIII only showed positive effects in one animal model. However, a pilot study conducted in a group of 10 patients with recurrent GBM (NCT01454596) showed that this therapy neither delayed tumor progression nor prolonged survival [150]. The trial failed, possibly due to the loss of EGFRvIII in recurrent tumors, similar to the ACT IV trial. A phase I clinical trial (NCT02208362) is currently underway to evaluate the safety and optimal dosing of CAR-T immunotherapy targeting the interleukin-13 alpha 2 receptor (IL13Rα2) for the treatment of patients with relapsed or refractory malignant glioma.

Results of clinical studies described a case of 50-year-old patient who underwent standard therapy, including tumor resection, radiotherapy and TMZ. However, after 6 months, magnetic resonance imaging (MRI) and positron emission tomography combined with computed tomography (PET-CT) of the brain showed signs of relapse. The patient received CAR-T cells directed against IL13Rα2, which led to a regression of the tumor. The clinical effect lasted for 7.5 months [151]. However, the recurrent tumor showed low expression of IL13Rα2, and therefore low target protein for CAR-T cells, and this is a major challenge for the use of this therapy in clinical practice. The studies on single-epitope CAR- T-cell therapy have not produced the expected results. However, the development of this therapy can be improved by using multivalent CAR-T cells targeting different glioma antigens. This approach can overcome the problem of antigen variability and tumor escape from the immune system [152]. Combining CAR-T therapy with immunotherapy using monoclonal antibodies can improve the immune system's ability to overcome GBM. Currently, a phase I clinical trial (NCT04003649) is recruiting patients to evaluate the efficacy of IL13Rα2 CAR-T cells alone or in combination with nivolumab and ipilimumab for the treatment of relapsed or refractory GBM.

B7-H3 (CD276) is a checkpoint protein. Its expression was found in tumour cells and immune cells from the tumour microenvironment [153]. B7-H3 is also associated with cancer cell proliferation, metastasis and treatment resistance, and therefore is a promising molecular target for CAR-T cells in GBM [153]. It is expressed in approximately 70 % of GBM patients, with two isoforms present in GBM and only one in healthy brain tissue: 2IgB7-H3 and 4IgB7-H3, respectively. Research has shown that recurrent GBM is characterized by increased expression of 2IgB7-H3, leading to resistance to TMZtreatment. The protein 4IgB7-H3, which is only found in cancer cells, may be a promising candidate for targeted GBM therapies, while 2IgB7-H3 may be associated with tumor resistance to chemotherapy [154]. Two phase I and I/II clinical trials (NCT05241392, NCT04077866) are currently underway to evaluate the safety and efficacy of B7-H3-targeted CAR-T therapy. The studies will also determine the maximum tolerated dose (MTD) and the recommended Phase II dose (RP2D). In the case of a Phase I/II trial, the efficacy of the therapy between TMZ cycles compared to TMZ alone will also be evaluated.

### CAR-NK

The hope for a possible breakthrough in GBM therapy also lies with NK cells. Unlike T-cell therapy, NK-cell therapy does not require the presence of specific tumor-associated surface antigens. This overcomes the problem of heterogeneity within and between GBM tumors, which limits the efficacy of CAR-T [155]. Natural killer (NK) cells identify healthy cells by detecting the presence of major histocompatibility complex (MHC) class I molecules. In contrast, cancer cells are identified by their reduced or absent expression of MHC-I. The receptor KIR (killer-cell immunoglobulin-like receptor) on the surface of NK cells binds to MHC-I, inhibits its function and prevents the destruction of healthy cells [156]. The activity of NK cells is regulated by the balance between signals from stimulatory and inhibitory receptors on their surface. These immune receptors enable the cells to identify and eliminate cancer cells [157]. In addition, NK cells play a crucial role in the recruitment of key cells for antitumor immunity, such as conventional dendritic cells type 1 (cDC1) and cytotoxic T lymphocytes. This helps to overcome the immunosuppressive tumor environment [158].

NK cells have properties that make them a promising therapeutic strategy in the medication against treatment-resistant cancers, including GBM. It is frequently observed that the number and activity of NK cells decreases in individuals diagnosed with cancer. In clinical trials of NK cell immunotherapy, the NK-92 cell line isolated from the blood of a 50-year-old man with malignant non-Hodgkin's lymphoma is used [159]. The cells of this line exhibit cytotoxic activity, produce cytokines and have unlimited proliferation potential. In addition, they can be genetically modified to express chimeric antigen receptors, resulting in CAR-NK cells that resemble CAR-T cells [160]. The ongoing phase I clinical trial (NCT03383978) aims to evaluate the safety and tolerability of HER2-specific CAR-NK cells 92/5.28.z in patients with relapsed or refractory HER2-positive glioma. This is due to the fact that elevated levels of this protein have been found in a significant percentage of GBM tumors [161]. The aim of the CAR2BRAIN study was to determine the maximum tolerated dose (MTD) and assess the safety of the therapy. In this study, participants were administered up to twelve injections of NK-92/5.28.z cells. The distribution of the injected cells in the brain, cerebrospinal fluid and blood was examined and their pharmacokinetics and pharmacodynamics were determined. The results suggest that immunotherapy based on NK cells in combination with other regimes of anticancer therapy could be a promising research option and a potential breakthrough in the treatment of GBM.

### Liposomes

In addition to immunotherapy, the use of nanotechnology, in particular liposome technology, is also promising for GBM therapy. Liposomes are small vesicles that consist of one or more lipid bilayers and serve as a non-toxic and biodegradable carrier for hydrophobic and hydrophilic drugs. In addition, their phospholipid structure is biocompatible with the BBB [162]. Liposomes have the advantage that they can be targeted by binding ligands for receptors present on the target cells to their surface.

A phase I clinical trial (NCT03603379) investigated the efficacy of immunoliposomes targeting the epidermal growth factor receptor (EGFR) when administering doxorubicin to GBM. The study was conducted in a group of patients with relapsed or refractory glioblastoma multiforme with amplification of the EGFR gene. Immunoliposomes containing doxorubicin (C225-ILs-dox) were administered intravenously to patients at a dose of 50 mg/m2 for up to four treatment cycles of 28 days each. The drug concentration in blood plasma, cerebrospinal fluid (CSF) and tumor tissue was then examined. The results showed that doxorubicin was detectable in the resected tumor, but not in the CSF. It is assumed that C225-ILs-Dox cannot overcome the intact BBB. However, in GBM, the BBB is often disrupted, which may allow the use of liposomes for drug delivery to tumor tissue.

The use of liposomes with ligands for receptors associated with the BBB has already been explored. The BBB is the main obstacle to the delivery of anticancer drugs to central nervous system tumors, including GBM. Key receptors associated with the BBB include the low-density lipoprotein receptor (LDLR), transferrin receptor (TfR) and glucose transporter (GLUT) [163]. In addition, cancer cells are characterized by an increased expression of TfR. This is due to their high demand for iron, which is necessary to maintain a rapid growth and proliferation rate [164]. To eliminate the BBB and achieve precise delivery of macromolecular drugs to the GBM, the researchers developed cationic liposomes to which a fragment of a single-chain anti-TfR antibody was attached. These liposomes also contained a plasmid with the human tumor suppressor gene TP53, which encodes wild-type p53 (SGT-53) [165]. In a phase II clinical trial (NCT02340156), the efficacy of treatment with TMZ in combination with tumor-targeted SGT-53 liposomes was investigated in patients with relapsed GBM. However, due to the small number of patients and the inability to perform a meaningful statistical analysis, SGT-53 with anti-TfR antibody did not pass phase II clinical trials.

An ongoing phase I/II clinical trial aims to determine the maximum tolerated dose/recommended dose of liposomal curcumin in combination with standard radiotherapy and TMZ in patients with newly diagnosed high-grade glioma (HGG) (NCT05768919). Curcumin, a plant polyphenol, exhibits anticancer properties by disrupting metabolic pathways and inhibiting angiogenesis in cancer cells [166]. The use of curcumin in free form in therapy is limited due to its low solubility in water and poor bioavailability. To improve its delivery to cancer cells, a carrier is necessary. In this case, liposomes are used as carriers. The study aims to include a group of about 30 patients receiving weekly intravenous infusions of liposomal curcumin. The efficacy of the treatment will be assessed by overall survival (OS) and progression-free survival (PFS) observed at three different doses of the drug. The duration of treatment for each patient is expected to be up to 34 weeks.

The liposomal form of verteporfin (Visudyne) is being tested in a phase I/II clinical trial for the pharmacologic control of GBM in patients with recurrent EGFR-positive GBM (NCT04590664). Verteporfin is an FDA-approved drug used as a photosensitizer for photodynamic therapy (PDT) of eye diseases. This therapy generates reactive oxygen species (ROS) by activating verteporfin with red light (635 nm) to eliminate abnormal tissue [167]. In vitro results have shown that verteporfin could also be used in the treatment of GBM. PDT with verteporfin as a photosensitizer caused cell death in human glioma stem cells. Verteporfin is a compound that can be internalized in GBM cells. It can be used for PDT and as a fluorescent dye for intraoperative tumor mapping [168]. The current clinical trial consists of two phases: Phase I aims to evaluate the safety and MTD of verteporfin, while Phase II aims to determine its antitumor activity by evaluating OS and PFS. Patients enrolled in the study will receive weekly intravenous infusions of verteporfin over 83 min for 6 weeks in Cycle 1. They will then receive weekly infusions for 5 weeks unless disease progression or significant toxicity occurs. The cycles are repeated every 6 weeks. After completion of therapy, patients are observed for 30 days, followed by observation every 12 weeks.

## Biomaterials as drug carriers

In silico models of GBM suggest three main features that could serve as targets for precision chemistry in this type of neoplasm: Angiogenesis, invasiveness and diffusion of GBM cells if they spread in brain tissue [169]. Computational modeling can be useful in predicting the efficacy of therapies in terms of the response of the tissue microenvironment [169]. In addition, in silico modeling is a tool for developing nanomedicine solutions and predicting their efficacy. New drug formulations, such as nanoparticles, have a significant impact on pharmacokinetic parameters and can be used to develop combination therapies with more appropriate patterns of drug concentration changes and BBB permeability properties. For example, drug encapsulation solutions such as delphinidin can improve the efficacy of drug delivery in brain tissue [169].

One of the new strategies for GBM treatment involves the use of nanomaterials and polymer carriers in which active drugs are embedded. For example, Gliadel® wafers (manufactured by Eisai Inc. for Azurity Pharmaceuticals, Inc.) containing carmustine can be implanted into the niche after resection of the GBM tumor. This strategy was approved by the FDA (U.S. Food and Drug Administration Agency) in 2003 as an alternative in GBM treatment, especially in combination with radiotherapy and TMZ, which increased the overall survival of patients by up to 4 months [170]. The main advantage of this approach is the biodegradation of the polymer, which releases carmustine slowly over two weeks [43]. However, clinical studies have shown that complications such as brain swelling, seizures, wound healing disorders or brain infections can occur in some cases, which could be a reason for excluding this treatment option.

New strategies for local or systemic application in GBM currently include polymer carriers such as nanoparticles, polymeric micelles, lipid-based drug delivery systems (liposomes) or hydrogels with the potential to incorporate active macromolecules [171]. It is worth noting that locally implanted treatments can bypass physiological barriers such as the BBB and can act more aggressively and directly on tumor tissue [171].

In addition to chemotherapy for GBM, other treatment options are being investigated, including EGFR inhibitors. One such inhibitor, afatinib, has been shown to extend patient survival in combination with TMZ. New EGFR inhibitors, such as erlotinib and gefitinib, are currently being investigated in clinical trials [172]. In the treatment of GBM, a combination therapy of TMZ and either BVC or afatinib has been shown to improve patient survival. However, the development of resistance to TMZ or radiotherapy remains a major challenge in GBM treatment, leading to poor outcomes with targeted anti-angiogenic or anti-EGFR therapies. BBB permeability is a significant obstacle to targeted drug delivery and the second mechanism of chemoresistance plays a crucial role in treatment failure [173]. New drug formulations such as liposomes, polymers and nanoparticle carriers are being investigated for their improved properties in targeted drug delivery against GBM. An in vitro study showed that the combination of doxorubicin and erlotinib encapsulated in liposomes improved the uptake of the drug by glioblastoma U-78 MG cells, which could be a promising alternative [174].

Studies on the encapsulation of TMZ, the most effective drug in GBM since 2005, have also been presented, although they are limited in number and efficacy has not been fully confirmed. TMZ carriers such as liposomes, solid lipid nanoparticles, polymeric nanoparticles, dendrimers, mesoporous silica nanoparticles, graphene oxide nanoparticles, and magnetic nanoparticles have been investigated to date [175]. These probes are necessary due to the limitations of TMZ, including hydrolysis, poor solubility, non-specific toxicity, and also due to GBM characteristics such as differentiation into a chemoresistant phenotype and the general heterogeneity of this neoplasm, not disregarding the permeability of the BBB [175].

### Electrical field

Optune's TTF (tumor treatment fields) devices (manufactured by Novocure) were approved by the FDA in 2015 as the latest innovation for the treatment of newly diagnosed GBM. This method applies electric fields with low intensity (1–3 V/cm) and medium frequency (200 kHz) pulses [176]. Despite the increase in disease-free survival, this method is not standard of care due to the marginal changes in overall survival, high cost and inconvenience to patients (skin toxicity and seizures) [177]. Remarkably, co-therapy with TMZ, RT and TTF has been observed to prolong progression-free survival by up to 56 % at six months in clinical trials conducted (NCT00916409). To date, this is the last FDA-approved therapy for GBM.

### Overcoming radiotherapy

Radiotherapy is still one of the standard therapies in the treatment of GBM, in addition to chemotherapy, usually TMZ [178]. However, the restrictions on the use of radiotherapy depend on the patient's general physical condition. The standard regimen involves radiotherapy over a period of 6 weeks, with a total dose of 40–60 Gy, usually in 2 Gy doses [179,180]. However, there is still a need to improve the effectiveness of γ-radiation exposure in the postoperative cavity to protect critical organs at risk such as optic nerves, optic chiasm, eyes, lenses, brain and brainstem with respect to the accepted margin [179]. Cheng et al. (2022) [181], have recently described an improvement in magnetic resonance imaging (MRI) that allows differentiation between neoplasms and normal brain tissue based on the protein VCAM-1 (vascular cell adhesion molecule-1). The higher expression of this protein in tumor endothelial cells as opposed to normal tissue could have the potential to better mark the interface between them. These results suggest a new role for the membrane protein in the treatment of GBM. In addition, the resistance to therapy in the development of GBM could be due to the reduced sensitivity of γ-irradiation. This phenomenon is related to the population of glioma stem cells (GSC) that survive radiotherapy and are involved in the recurrence of the radioresistance phenotype. GSC are thought to be the main reason for GBM tumor recurrence [182]. Mechanistically, increased expression of N-cadherin is involved in radioresistance, which inhibits the Wnt/β-catenin signaling pathway and causes anti-apoptotic effects and decreased neuronal differentiation. The observed increase in N-cadherin levels was caused by IGF-1, which was upregulated by γ-irradiation. Picropodophyllin, a clinically approved IGF-1 inhibitor, may have the potential to reverse the radioresistance phenotype of GBM [183]. In this regard, targeted N-cadherin therapy could be a promising direction to combat this aggressive phenotype.

### Alteration of the lipid metabolism

Lipid metabolism is significantly altered in glioblastoma, not only limited in glioma itself, but present in the whole brain. Changes in lipids are frequently observed in neoplasms and also in other neuronal disorders, in autoimmune diseases such as multiple sclerosis, have also been described [[184], [185], [186]]. A typical finding in GBM diagnosis is an increase in choline levels observed on MRI, which reflects an imbalance in lipid metabolism. Changes in lipidomics and fatty acids appear to be a promising area for GBM treatment. Fatty acids are involved in the induction of immune scavenging in GBM, promote cell proliferation and also regulate ferroptosis [187,188].

First, as a way to combat neoplasms by destabilizing metabolism through an imbalance of anabolism and catabolism processes in GBM cells. Secondly, lipid droplets serve as a space for drug delivery and changes in lipid droplet content can attenuate this phenomenon. Oxidation of fatty acids, especially β-oxidation, is a potential target in anti-GBM therapy. However, this is a relatively new area of research and so far only two clinical trials (NCT05118776 and NCT03032484) have been conducted using fatty acid synthase as a target protein, with inhibitors: TVB-2640 and ASC40. The detailed role of fatty acids in GBM has been described in an excellent review by Jason Miska and Navdeep S. Chandel [189]. Recently, CDKN2A was also found to be responsible for the distribution of polyunsaturated fatty acids (PUFA) in lipid droplets. As observed in an in vitro and in vivo model of GBM, the consequences of CDKN2A deletion were lipid peroxidation and ferroptosis, resulting in prolonged survival of xenografts in mice. These findings open up new possibilities for the reorganization of lipid metabolism in GBM and also for the development of glutation peroxidase inhibitors suitable for brain treatment [190].

### Ferroptosis

Ferroptosis leads to an accumulation of lipid peroxide and thus to non-apoptotic and non-necrotic cell death due to an iron-dependent mechanism [191]. Lipid metabolism is closely linked to ferroptosis. ROS generated in the Fenton reaction, in which Fe^2+^ is converted to Fe^3+^ in the presence of H_2_O_2_, are involved in the peroxidation of PUFAs in cell structures such as membranes and alter them, leading to cell death [238]. Recently, the usefulness of ferroptosis in anticancer treatment, especially in GBM, has been the focus of research [192]. Ferroptosis induction, including by disulfiram, resulted in recurrence of radiotherapy sesitivity in vitro studies on glioblastoma cell lines U251 and LN229 [193]. It is worth noting that mutation of p53 determines the response to pro-ferroptosis treatment in GBM, while this response is weaker in p53 wild-type GBM in an in vivo mouse model [194].

One of the latest concepts of ferroptosis useful in targeting GBM is found in the work of Cao and coworkers, in which the authors described the anti-tumor effect of biomimetic nanoparticles consisting of macrophage membrane-coated nanoparticles as carriers for the ferroptosis inducer ALOX15 lipoxygenase in a mouse model of GBM [195]. Induction of ferroptosis could also be triggered by fatostatin and acts mechanistically by inhibiting the AKT/mTORC1/GPX4 pathway in GBM and has anti-tumor effects in vitro and in vivo models [196]. In addition, recent studies have shown that decreased ferroptosis is related to the TMZ-resistant phenotype of GBM, and to date, the Fanconi anemia-associated gene Fanconi anemia complementation group D2 (FANCD2) has been found to play this role [197].

Moreover, ferroptosis is linked to apoptosis and autophagy, and this interplay is observed in GBM, which is considered a potential approach for therapeutic strategies, with many questions still open that deserve a scientific answer [198].

### Autophagy

Autophagy is a lysosome-mediated process that is observed at different stages of carcinogenesis as a dual tumor-promoting or tumor-suppressing mechanism, depending on tumor microbiota formation. In general, this process increases cancer cell survival during unfavorable times such as starvation [199]. Autophagy is also observed in GBM as a side effect of TMZ treatment and is responsible for the chemoresistant phenotype of GBM. Some autophagy inhibitors, such as chloroqiunone, cause these neoplasm cells to become resistant to TMZ without significantly increasing the overall survival rate of patients [152]. In contrast, the antitumor role of autophagy, classified as exosomal or secretory autophagy, has been described in GBM and associated with repolarization of macrophage M1 to a tumor-suppressive M1 phenotype, resulting in TMZ sensitization [200]. In more recent studies by Chryplewicz et al. [199], CD8+ and CD4+ *T* cell efflux was observed as a result of stimulation of autophagy following concomitant treatment with VEGFR inhibitors and the tricyclic antidepressant imipramine. Surprisingly, this stimulation of autophagy also leads to an increase in PDL-1 and together suggests a potential for immunotherapy in GBM in conjunction with autophagy inducers [201]. In addition, autophagy inducers such as loperamide, pimozide and STF-62,247 were found to induce cell death in vitro in MZ-5426,29, LN-229 and U343 GOS-3 glioblastoma cells through large-scale induction of autophagy [202]. Currently, no autophagy inducers are clinically viable, and deeper insights are urgently needed to better understand the potential of autophagy-based therapy in GBM.

## Concluding remarks and future challenges

GBM is one of the most vascularized tumors, with a dynamic molecular differentiation in terms of chemoresistance and a γ-resistant phenotype that emerges during treatment. Targeted therapies have not yet produced successful results, despite the administration of BVC, which was believed to be a milestone for clinical outcomes, but it does not improve overall patient survival alone. To improve clinical outcomes of GBM patients, therapeutic strategies targeting different goals should be considered when developing new treatments. GBM therapy is undoubtedly one of the greatest challenges in the field of oncology. This review presents some current multidisciplinary solutions that could be seen as the beginning of new possibilities in the design of GBM treatment. On closer inspection, the mechanisms involved in GBM development and progression are interconnected and overlapping, and a holistic approach could yield the most clinically meaningful new co-therapy regimens. New drug carriers, such as lisosomes and polymers, are predestined to bypass the BBB and should therefore be considered in the development of therapeutic strategies.

## CRediT authorship contribution statement

**Agnieszka Rusak:** Writing – review & editing, Writing – original draft, Conceptualization. **Benita Wiatrak:** Writing – review & editing, Writing – original draft. **Klaudia Krawczyńska:** Writing – review & editing, Writing – original draft. **Tomasz Górnicki:** Writing – review & editing. **Karol Zagórski:** Writing – review & editing. **Łukasz Zadka:** Writing – review & editing. **Wojciech Fortuna:** Supervision.

## Declaration of competing interest

The authors declare that they have no known competing financial interests or personal relationships that could have appeared to influence the work reported in this paper.
